# Circular bioeconomy-driven carotenoid production by *Agrococcus* sp. NP24 using cheese whey byproduct: process optimization and bioactivity assessment

**DOI:** 10.3389/fmicb.2025.1747717

**Published:** 2026-02-03

**Authors:** Nehad Noby, Fatma Elsayed, Mahmoud M. Agami, Nadia A. Soliman

**Affiliations:** 1 Biotechnology Department, Institute of Graduate Studies and Research (IGSR), Alexandria University, Alexandria, Egypt; 2 Medical Research Institute (MRI), Alexandria University, Alexandria, Egypt; 3 Research and Innovation Hub, Alamein International University, Alexandria, Egypt; 4 Bioprocess Development Department, Genetic Engineering & Biotechnology Research Institute (GEBRI), City of Scientific Research & Technological Applications (SRTA-City), Alexandria, Egypt

**Keywords:** *Agrococcus* bacteria, anticancer, antioxidant, carotenoids, cheese whey, circular economy, waste valorization

## Abstract

Growing interest in the circular economy has promoted the use of agri-food wastes as fermentable and readily available substrates for microbial cultivation, offering a sustainable and cost-effective strategy for natural pigment production. In this study, cheese whey was utilized as a nutritional substrate for pigment synthesis by an isolated strain identified as *Agrococcus* sp. NP24 (PQ097720.1). The work further aimed to characterize the produced pigment and evaluate its bioactivity. The culture medium was optimized using a Box–Behnken design (BBD). The carotenoid profile of the extracted pigment was analyzed by HPLC-DAD and LC–MS. Pigment stability was assessed across a range of pH values and temperatures, and its antimicrobial, antioxidant, and anticancer activities were examined. The pigment was identified as zeaxanthin monoester (C14:0). The maximum pigment yield (0.0567 mg/mL extract) was achieved after 72 h at 20 °C using a medium containing 80% whey (v/v), 0.5% peptone (w/v), 0.97 g % casein (w/v), and supplemented with 0.5% (w/v) yeast extract and 0.5% (w/v) MgSO₄. The pigment remained fully stable up to 50 °C. Acidic conditions (pH 3–5) enhanced pigment absorbance compared to neutral and alkaline pHs. In contrast, exposure to daylight markedly reduced pigment stability, leaving only 26% residual activity after 1 h. The pigment exhibited potent antioxidant activity with an IC₅₀ of 6 μg/mL. It also showed cytotoxic and significant selectivity against the triple-negative breast cancer cell line MDA-MB-231 and the colorectal carcinoma cell line HCT-116, with IC₅₀ values of 3.3 mg/mL and 0.56 mg/mL, respectively, while no cytotoxicity was observed toward the HepG-2 hepatoblastoma cell line. The carotenoid did not display significant antimicrobial activity. In conclusion, the cost-effective production of NP24 carotenoids, combined with their favorable stability and bioactivity, supports their potential use as natural colorants in food applications.

## 1 Introduction

Rising the world concern of environmental and health impacts of synthetic dyes has urged the need of natural, safe and ecofriendly alternatives. Traditionally, synthetic dyes have provided the sustained demand of colorants in food, pharmaceutical and textile industries. Their availability, low cost and stability have underpinned their preference for bright and stable coloration (Negi, 2025). However, recent studies have proved an association between synthetic food colorants and many health concerns (Barciela et al., 2023; Vieira Rubio et al., 2025; Alnuqaydan, 2024).

Numerous environmental fears have been also evolved regarding the extensive use of synthetic dyes. In addition to contaminating the human food chain, the leakage of dye effluents into water bodies reduces light penetration, thereby inhibiting photosynthetic activity in aquatic plants, disrupting the microbial balance and affecting soil fertility (Taha and Gouda, 2025; Ali, 2010). Furthermore, several synthetic colorants and their degradation products are known to be mutagenic, carcinogenic, or resistant to biodegradation, posing long-term ecological and health risks (Ali, 2010; Fried et al., 2022).

Although plant dyes are used as colorants in old human civilizations (Fried et al., 2022), their large-scale use is restricted by their low pigment yield, seasonal variations, and the competition for arable lands and water resources. Moreover, many plant dyes exhibit poor stability under light, temperature and pH variations (Lu et al., 2023), which limits their applicability in cosmetic and food products. The extraction of plant pigments is often inefficient and relies on a combination of organic solvents, thereby raising both economic and environmental concerns (Pati and Sarkar, 2022). These limitations highlight the necessity for potent alternative natural colorants.

Microorganisms have emerged as a potent sustainable source for natural biodegradable pigments. Several species of bacteria, yeast and fungi produce pigment to serve certain biological roles in microorganisms, such as the control of defense mechanisms and microbial interactions, which might explain their antimicrobial activity (Kirti et al., 2014). In other cases, they contribute the survival of the producer strains under harsh condition such as photooxidative damage caused by solar radiation due to their significant antioxidant qualities (Reis-Mansur et al., 2019). Microbial pigments can also be secreted conditionally in a stress-dependent manner, particularly under conditions such as high salinity or low temperature, to maintain the optimal fluidity and permeability of cellular membranes (Flegler and Lipski, 2021; Li et al., 2023).

Microorganisms produce pigments in different elegant shades with broad stability at different pH and temperatures (Saubenova et al., 2024). Various classes of microbial pigments have been identified, including flavins, carotenoids, monascin, anthraquinone and melanin, each characterized by distinct biosynthetic pathways and functional properties (Afroz Toma et al., 2023; Saubenova et al., 2024). Beyond their natural color, most of the tested pigments have demonstrated wide bifunctionalities like antioxidant and anti-inflammatory, making them appealing candidates as functional food additives and active ingredients in pharmaceutical products (Saubenova et al., 2024; Barreto et al., 2023).

Carotenoids represent the most widely distributed pigments in nature, produced in yellow, orange and red hues. They are produced in several microorganism-like bacteria, fungi, archaea and algae, in addition to their synthesis in some plants that appear in bright red-yellow colors. On the other side, animals like insects, crustaceans cannot synthesis them *de novo* while they mainly gain them through their diet (Maoka, 2020).

Structurally, most carotenoids harbor a polyene chain with nine conjugated double bonds arranged in a C40 skeleton produced from the condensation of two C20 geranyl-geranyl diphosphate chain in a head to tail manner (Siziya et al., 2022). Other less common carotenoids, synthesized in shorter (C30) or longer (C50) skeleton are produced in rare types of microorganisms. For instance, *Staphylococcus aureus* synthesizes a C30 golden-yellow pigment, known as staphyloxanthin, that helps in oxidative stress relief. On the other hand, C50 carotenoid known as bacterioruberin can be produced in some halophilic and psychrotolerant strains to maintain membrane functionalities (Flegler and Lipski, 2021; Pelz et al., 2005; Noby et al., 2023).

Despite the continuously expanded popularity of microbial pigments because of their safety, biodegradability and natural aesthetic colors, the sustainable production on the commercial scale has not been achieved yet. The nutritional medium cost is one of the major obstacles hindering the economic scale production of microbial pigments. To overcome this challenge, considerable efforts have been considered to employing low-cost substrates as nutrient sources for microbial growth (Grewal et al., 2022). Agricultural and food processing residues are rich in proteins, carbohydrates, vitamins, and minerals. However, the improper disposal of agricultural and food residues poses serious environmental challenges such as greenhouse gas emission, soil and water pollution and proliferation of pathogens (Yaashikaa et al., 2022). Thus, the valorization of these residues into bio-based compounds, particularly natural pigments, provides an environmentally sustainable approach to addressing the growing need for bio-colorants and promoting the circular economy framework (Banu et al., 2021).

The circular economy concept seeks to reduce waste and enhance resource efficiency by prolonging the lifecycle of materials inside production processes. Unlike the classic model of “take–make–dispose,” the circular approach highlights recycling, reuse, and bioconversion of waste into valuable products. The conventional linear production systems rely on refined substrates and energy-intensive waste management; however, circular bioeconomy-based fermentation integrates waste streams directly into the production cycle, thereby reducing capital investment associated with raw material purification, effluent treatment and disposal (Zaky et al., 2022). In addition, the use of minimally processed by-products such as whey or other nutrient-rich residues lowers the overall energy demand by eliminating upstream processing steps and enabling biological conversions into valuable products under mild operating conditions (Pescuma et al., 2015). Through direct waste reutilization, and energy recovery from residual biomass, circular bioeconomy strategies significantly decrease both capital expenditure and energy consumption per unit product, enhancing the economic feasibility and sustainability of fermentation-based bioprocesses. Moreover, its low carbon economy concept supports the potential of more sustainable and greener environment (Khanna et al., 2024; Mohan et al., 2016).

In the context of microbial pigment production, agriculture and food residues can be used as regenerative feedstocks for microbial fermentation (Vural Gursel et al., 2022). These solutions not only mitigate the environmental impact of waste disposal but also enhance economic sustainability by transforming waste streams into economically viable bioresources.

Many agri-food byproducts and wastes such as, corn steep liquor, bran, whey, molasses fruit pomace, seeds, peels, etc. have been utilized as possible fermentable substrates to produce bio-pigments using bacteria, yeast and fungi. For instance, *Monascus ruber* Tieghem IOC 2225 had produced red pigment using corn steep liquor in a higher titer compared to glucose based- medium under the same conditions (Hilares et al., 2018), supporting the high nutritional value of complex medium compared to synthetic one. In another study, glycerol by-product was applied as a carbon source by *R. glutinis* TISTR 5159 for carotenoids production (Saenge et al., 2011). Potato peels were used in solo as nutritional medium for bio-pigments production using *Streptomyces* genus train SO6 with a yield of 1.75 mg/g (Schalchli et al., 2021).

The increasing emphasis on the circular economy framework in natural pigment production has driven efforts to expand agri-food wastes employment as renewable and fermentable substrates for microbial cultivation, offering a sustainable and cost-effective route for bio-pigment synthesis. In this respect, the present study demonstrates the successful application of the circular economy concept through the optimization of carotenoid production by a psychrotolerant *Agrococcus* strain using cheese whey as a readily available, low-cost substrate.

## 2 Materials and methods

### 2.1 Materials

Peptone, yeast extract, MgSO_4,_ casein, glycerol and agar were obtained from Biolife Co., Italy. Universal primers for 16S rRNA were synthesized by Willowfort, UK. Standard Master Mix 2X was purchased from Promega, USA. Methanol, absolute ethanol, *N*-methyl-dimethyl sulphoxide (DMSO) and sodium hydroxide (NaOH), were obtained from Piochem (Piochem Laboratory Chemicals), Egypt. 2,2-Diphenyl-1-picrylhydrazyl (DPPH) was obtained from Sigma-Aldrich Co., USA. Cheese whey was kindly provided from dairy processing department, faculty of Agriculture, Alexandria University.

### 2.2 Cell lines

Colorectal carcinoma HCT-116 cell line (RRID: CVCL_0291, ATCC^®^ CCL-247™), human hepatoblastoma HepG-2 cell line (RRID: CVCL_0027, ATCC^®^ Cat. No. HB-8065™), and human triple negative breast carcinoma (TNBC) MDA-MB-231 cell line (RRID: CVCL0062, ATCC HTB-26™). The three tested cell lines were obtained from Nawah Scientific, Egypt.

### 2.3 Growth and production media

Peptone–yeast (PY) medium was used for the isolation and routine activation of the bacterial strains. The medium consisted of 10 g L^−1^ peptone, 5 g L^−1^ yeast extract, and 5 g L^−1^ NaCl. Peptone–yeast agar (PA) medium was prepared by supplementing PY medium with 1.5% (w/v) agar.

A partially optimized medium obtained from the OVAT experiment consisted of 50% (v/v) cheese whey, 10 g L^−1^ peptone, 5 g L^−1^ casein, 5 g L^−1^ yeast extract, and 5 g L^−1^ MgSO₄.

The fully optimized medium consisted of 80% (v/v) cheese whey, 5 g L^−1^ peptone, 0.979 g L^−1^ casein, 5 g L^−1^ yeast extract, and 5 g L^−1^ anhydrous MgSO₄.

### 2.4 Methods

#### 2.4.1 Isolation and identification of carotenoid producing strain

Isolation of pigmented bacterial strains was performed from soil samples. The collected soil samples were activated in 50 mL peptone yeast medium (PY) for 18 h., the activated culture was then serially diluted on Petone yeast agar medium (PA), followed by incubation for 48 h. at 20 °C. The developed pigmented colonies were picked up, purified and preserved at −20 °C on 40% glycerol. The selected bacterial strain was examined morphologically and further identified by molecular characterization using 16S rRNA gene sequencing (Weisburg et al., 1991). Genomic DNA was extracted from a pure culture of the selected strain using DNA miniprep kit (D3024, ZYMO), according to manufacturer’s instructions. The primer pair s F27/R1429 was used to amplify the 16 S rRNA universal gene (Weisburg et al., 1991). The resulting nucleotide sequence was analyzed via BLASTn tool provided by the NCBI. Finally, phylogenetic analysis was carried out using MGEA 9 software with closely related taxa to estimate the taxonomic affiliation of the isolate.

#### 2.4.2 Pigment production, extraction and quantification

The selected bacterial strain was activated on PA plate. The grown colonies were activated in 20 mL PY medium and incubated at 20 °C for 18 h. A volume of 5% of the grown culture was inoculated into 100 mL fresh PY medium prepared in 500 mL Erlenmeyer flask, the culture was incubated at 150 rpm, 20 °C for 3 days.

At the end of the incubation period, the culture was harvested via centrifugation at 1,677 × *g* for 10 min. Cell pellets were then suspended and soaked in methanol until completed bleaching. The extract was clarified by centrifugation at 5,662 × *g* for 10 min. to remove cell debris. The methanolic extract was further stored in dark conditions at −20 °C for subsequent analysis. The extracted pigment was quantified using the percentage extinction coefficient (E₁%^1^cm) method as reported previously (Liaaen-Jensen and Jensen, 1971).

#### 2.4.3 Structural characterization of the produced pigment

The spectral analysis of the extracted pigment was carried out through ultraviolet–visible (UV–Vis) spectrometry and Fourier Transform Infra-red (FT-IR) spectrometry. The UV–Vis spectrum of pigment methanolic extract was recorded using a Thermo Fisher Scientific spectrophotometer (Madison, WI, United States) over the range of 200–800 nm, with methanol as the blank. In FT-IR analysis, pigment methanolic extract was placed in a salt cuvette and analyzed using an FT-IR spectrometer (Perkin Elmer, Germany) over the range of 4,000–450 cm^−1^ to estimate the functional groups of pigment structure.

The extracted pigment was further analyzed using high-performance liquid chromatography (HPLC), coupled with diode array detection (LC-DAD) (AZURA Analytical (U) HPLC 862 bar System) and tandem mass spectrometry (LC–MS/MS) (Thermo Fischer Vanquish Horizon UHPLC system combined with TSQ Fortis™ Plus Triple Quadrupole Mass Spectrometer), a volume of 2 μL of the prefiltered pigment extract was injected into C_18_ reversed phase column (150 mm x 4 mm, 5 μm, Hypersil GOLD™, Lithuania) maintained at 30 °C. Separation of carotenoids was achieved using isocratic elution of 60% Acetonitrile in 0.1% Formic acid for 30 min.

#### 2.4.4 Stability test of pigment extract

The stability of the extracted pigment was evaluated under various physical conditions, including temperature, pH, and exposure to sunlight. A known concentration of pigment methanolic extract was incubated under each tested condition for 1 h. The residual pigment concentration was determined spectrophotometrically at the maximum absorption wavelength (λ_max = 435 nm), and the stability percentage was calculated according to the following Equation 1:


$$\text{Residual}\%=[{\left(\mathrm{Ct}/\mathrm{C0}\right)}^{*}\;100]$$ (1)

Where, C1 and C0 are the concentrations of teste sample and control sample, respectively, the statistical significance was set at *p* < 0.05.

For temperature stability, the pigment extract was incubated at different temperatures, ranging from 20 °C to 70 °C for 1 h. A non-incubated sample was used as the control.

For pH stability, equal volumes (1:1, (v/v)) of the pigment methanolic extract and 100 mM buffer solutions were mixed and incubated at 4 °C for 1 h. The following buffers were used: acetate buffer (pH 3.0–5.0), phosphate buffer (pH 6.0–7.0), and Tris–HCl buffer (pH 8.0). The buffer volume was replaced with methanol in the control sample.

For light stability, the test sample was exposed to natural daylight for 1 h., while a non-exposed sample served as the control.

#### 2.4.5 Optimization of whey-based medium for pigment production

A cheese whey–based medium was used as a cost-effective substrate for carotenoid production. The medium was formulated to contain 50% (v/v) cheese whey, 10 g L^−1^ peptone, 5 g L^−1^ yeast extract, and 5 g L^−1^ anhydrous MgSO₄. The culture medium (75 mL working volume) was prepared in 250 mL Erlenmeyer flasks. The initial pH was adjusted to 7.0, and incubation was carried out at 20 °C with agitation at 85 rpm for 3 days.

Pigment production was initially optimized using the one-variable-at-a-time (OVAT) approach, in which individual factors were varied while keeping all others constant. First, the effect of culture working volume on pigment yield was evaluated using different medium volumes (50, 75, 100, and 150 mL) prepared in 250 mL Erlenmeyer flasks. All cultures were incubated at 20 °C with agitation at 85 rpm for 3 days (statistical significance was set at *p* < 0.05).

The effect of medium composition was also examined through OVAT experiments. Specifically, potassium chloride (KCl) was supplemented into the production medium, and peptone was replaced with casein to qualitatively evaluate the influence of nitrogen source on pigment biosynthesis (statistical significance was set at *p* < 0.05).

Although the OVAT approach is useful for identifying significant individual factors, it does not account for possible interactions among variables. Therefore, the statistical optimization represented in response surface methodology will help in estimating the optimal concentration of the tested variables as well as the interactions among them (Reji and Kumar, 2022). In this context, three independent variables cheese whey (X1), peptone (X2) and casein (X3) were studied through Box Behnken Design (BBD) matrix to maximize carotenoid yield.

Each independent variable was represented in three coded levels, designated as −1 (low level), 0 (center level), and + 1 (high level), in a full design matrix of 15 experimental trials. The dependent variable (response) was expressed as carotenoids yield in (mg/mL). Each trial was prepared in 250 mL Erlenmeyer flasks with 50 mL medium, inoculated with 5% (v/v) of a 48 h preculture (~3 × 10^7^ CFU), and incubated at 20 °C with shaking at 85 rpm for 72 h.

The three independent variables were displayed by Equation 2:


$$\begin{array}{l} Y=\beta 0+\beta 1(\mathrm{X1})+\beta 2(\mathrm{X2})+\beta 3(\mathrm{X3})+\beta 12(X1\mathrm{X2})\\ +\beta 13(X1\mathrm{X3})+\beta 23(X2\mathrm{X3})+\beta 11(\mathrm{X1})\;2\\ +\beta 22(\mathrm{X2})\;2+\beta 33(\mathrm{X3}) \end{array}$$ (2)

Where *Y* represents the response, β0 is model intercept, β1, β2, and β3 are linear coefficients, β12, β13, and β23 are cross coefficients, while β11, β22, and β33 are quadratic coefficients.

For model validation and accuracy evaluation, the maximum yield predicted from the model was verified experimentally and the deviation percentage between predicted and achieved values was calculated.

#### 2.4.6 Biological activity of pigment extract

##### 2.4.6.1 Antimicrobial activity

The antimicrobial effect was assessed with 5 mg/mL of NP24 carotenoid dissolved in dimethyl sulfoxide (DMSO) against 0.5 McFarland of *E. coli* ATCC 25922, *S. aureus* ATCC 29213, and *C. albicans* ATCC 90028 using the agar well diffusion method (Perez, 1990). In this technique, the microbial strains were distributed separately on solidified medium plates, the wells are cut into solidified agar using a circular cork borer with 6 mm diameter. The wells were then filled with carotenoid solution (100 μL). Pure DMSO solvent served as the control test. The plates were first incubated at 4 °C for 1 h. to guarantee equal distribution of the extract, then incubated overnight at 37 °C. The zone of inhibition around the wells were compared with the control well.

##### 2.4.6.2 Antioxidant activity

The antioxidant efficiency of *Agrococcus* sp. NP24 was assessed spectrophotometrically at 517 nm through free radical scavenging activity (RSA) using 2,2 Di Phenyl Picryl Hydrazil (DPPH) (Marinova and Batchvarov, 2011). Different concentrations of the extracted carotenoid were tested to estimate the IC50 value (6, 3, and 1.2 μg/mL). The total samples volume was brought into 250 μL with ethanol and mixed with 1 mL of 70 μM DPPH solution, the reaction was incubated at 20 °C in dark. The scavenging efficiency was measured at intervals along 1 h. incubation time. The sample volume was replaced with ethanol in the control sample. The scavenging efficiency was calculated according to Equation 3:


$$\begin{array}{l} \%\text{Radical scavenging efficiency}\\ ={\left[(A_{\text{control}}-A_{\text{sample}})/A_{\text{control}}\right]}^{*}\;100 \end{array}$$ (3)

##### 2.4.6.3 Anticancer activity

###### 2.4.6.3.1 *In vitro* culture conditions

The three tested cell lines; HCT-116, HepG-2 cell line and MDA-MB-231 were cultured according to the American Type Culture Collection (ATCC) guidelines. Cultures were maintained using DMEM (Dulbecco’s modified Eagle’s medium), fortified with glucose (4.5 g/L) and L-glutamine (4 mM) (Lonza, United Kingdom). Complete culture medium preparation included the addition of fetal bovine serum (FBS) (MSE, France) at a concentration of 10% (v/v). Human peripheral blood mononuclear cells (PBMCs), as reference normal cells, were isolated from 5 mL of heparinized human blood, as described by Boyum (1968), using Histopaque^®^-1077 (Sigma–Aldrich, United States) density gradient separation medium, with the culture maintenance performed in complete Roswell Park Memorial Institute-1640 (RPMI-1640) medium (Lonza, United Kingdom). Incubation was carried out at 5 ± 1% CO2, 95 ± 5% humidity, and 37 ± 1 °C in a CO_2_ incubator (Nuaire, Germany).

###### 2.4.6.3.2 Evaluation of the ***in vitro*** anticancer effects of pigment extract

An MTT assay was adopted for this purpose as detailed by Vijayarathna and Sasidharan (2012). Cells were dispensed into 96-well plates (1 × 10^4^ cells/well) and further incubated under the previously described *in vitro* culture conditions for 24 h. Cells were treated with serial twofold dilutions of the carotenoid extract (5.6–0.043 mg/mL in complete culture medium) for 48 h after which the treatment medium was discarded followed by washing with saline solution (0.89%). Afterward, 100 μL of filtered MTT solution [0.5 mg/mL in phosphate buffer saline (pH 7.3–7.5) (Lonza BioWittaker, United Kingdom) was added]. Following a 2-h incubation, supernatants were carefully discarded, and 100 μL of dimethyl sulfoxide was used for solubilizing the precipitated formazan crystals before measuring the UV–Vis absorbance utilizing 96-well microplate spectrophotometer (Bio-Rad, Japan) at 490 nm.

An MTT assay was also employed to evaluate the extract’s effect on the cellular viability of normal PBMCs, following the method of Molaae et al. (2017). PBMNCs were dispensed as 100 μL (2 × 10^5^ cells) aliquots into a 96-well plate wells and treated with serial dilutions of the extract (33.5–4.2 mg/mL in RPMI-1640 complete culture medium). The assay was performed as described earlier under the same conditions adjusted to accommodate the suspended state of the PBMCs. Trials were in duplicate, and the anticancer activity was conveyed as the half-maximal inhibitory concentration (IC50) determined through regression analysis of dose–response curves using CompuSyn software (Chou and Martin, 2005).

### 2.5 Carotenoid extract selectivity

To assess the selectivity of the anticancer attribute of the carotenoid extract from *Agrococcus* sp., the selectivity index (SI) value was determined as the ratio of the extract IC50 value recorded for the PBMCs to the extract IC50 value recorded for each cell line, separately, as established by Nogueira and do Rosário (2010).

## 3 Results

### 3.1 Isolation and identification of carotenoid producing bacterial strain

**Figure 1:**
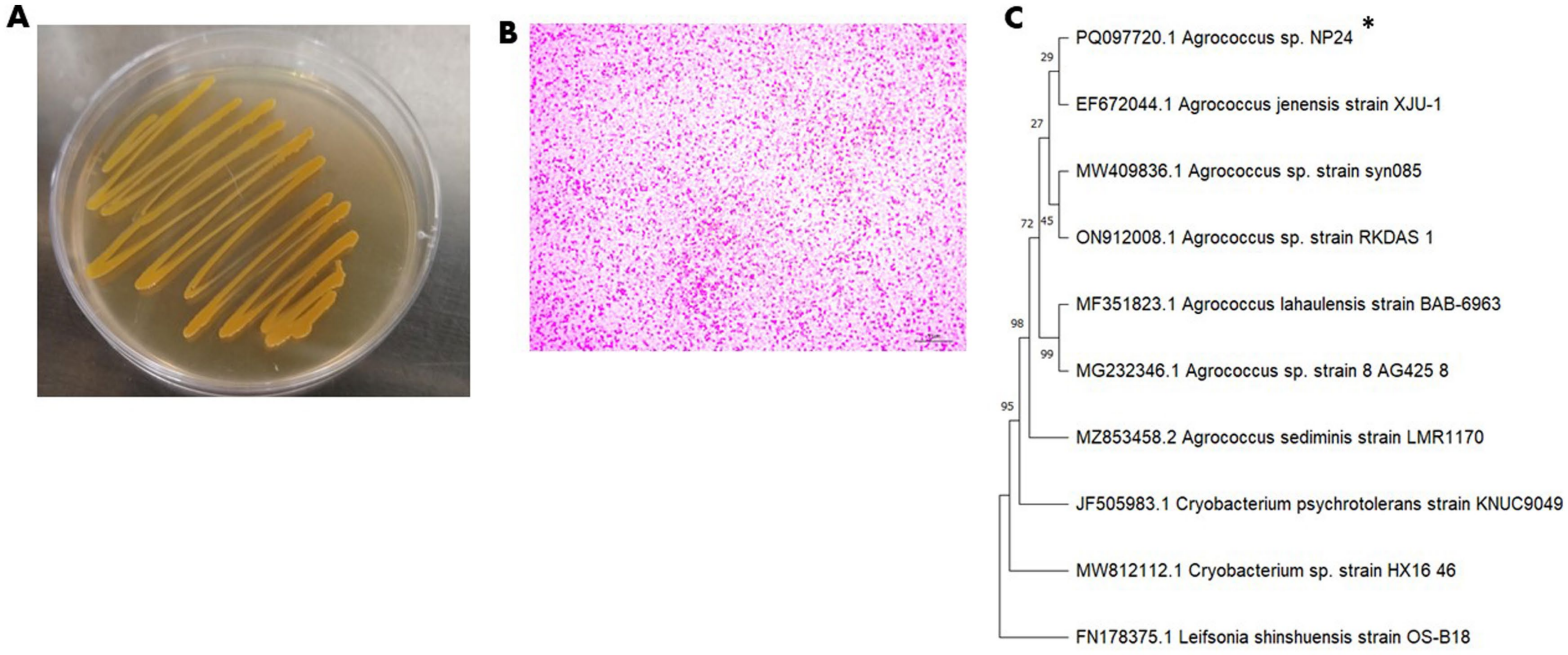
Phenotypic and molecular identification of the isolated bacterial strain. **(A)** Colony morphology of *Agrococcus* sp. NP24 strain on PY medium. **(B)** Cell morphology under light microscope showing irregular rod shape cells. **(C)** Phylogenetic relatedness between NP24 strain sequence and closely related bacteria using neighbor joining method, *Leifsonia shinshuensis* is used as an outgroup. Accession number is indicated for each genus. Bootstrap values are represented as a percentage of 500 replicates.

Microbial isolation yielded a yellow-pigmented bacterial strain. On PA medium, the colonies appeared smooth, yellow, and rounded (Figure 1A), with a noticeable darkening of color after storage at 4 °C. The cells were Gram-positive and appeared in short irregular rods under the light microscope (Figure 1B).

The BLASTn analysis of 16S rRNA sequence showed high similarity to several closely related species of genus *Agrococcus* genus. The highest sequence identities (≥99%/) were observed with species *Agrococcus jenensis* (accession no. EF672044.1) *A. lahaulensis* (accession no. MF351823.1) and *A. sediminis* (accession no. MZ853458.2). Phylogenetic analysis of the 16S rRNA gene sequence and comparison with related sequences revealed that the isolate belongs to the genus *Agrococcus* within order actinomycetales. The strain was designated *Agrococcus* sp. NP24 (Figure 1C), and its 16S rRNA sequence was deposited in GenBank under the accession number PQ097720.1.

### 3.2 Identification and structural characterization of the produced pigment

**Figure 2:**
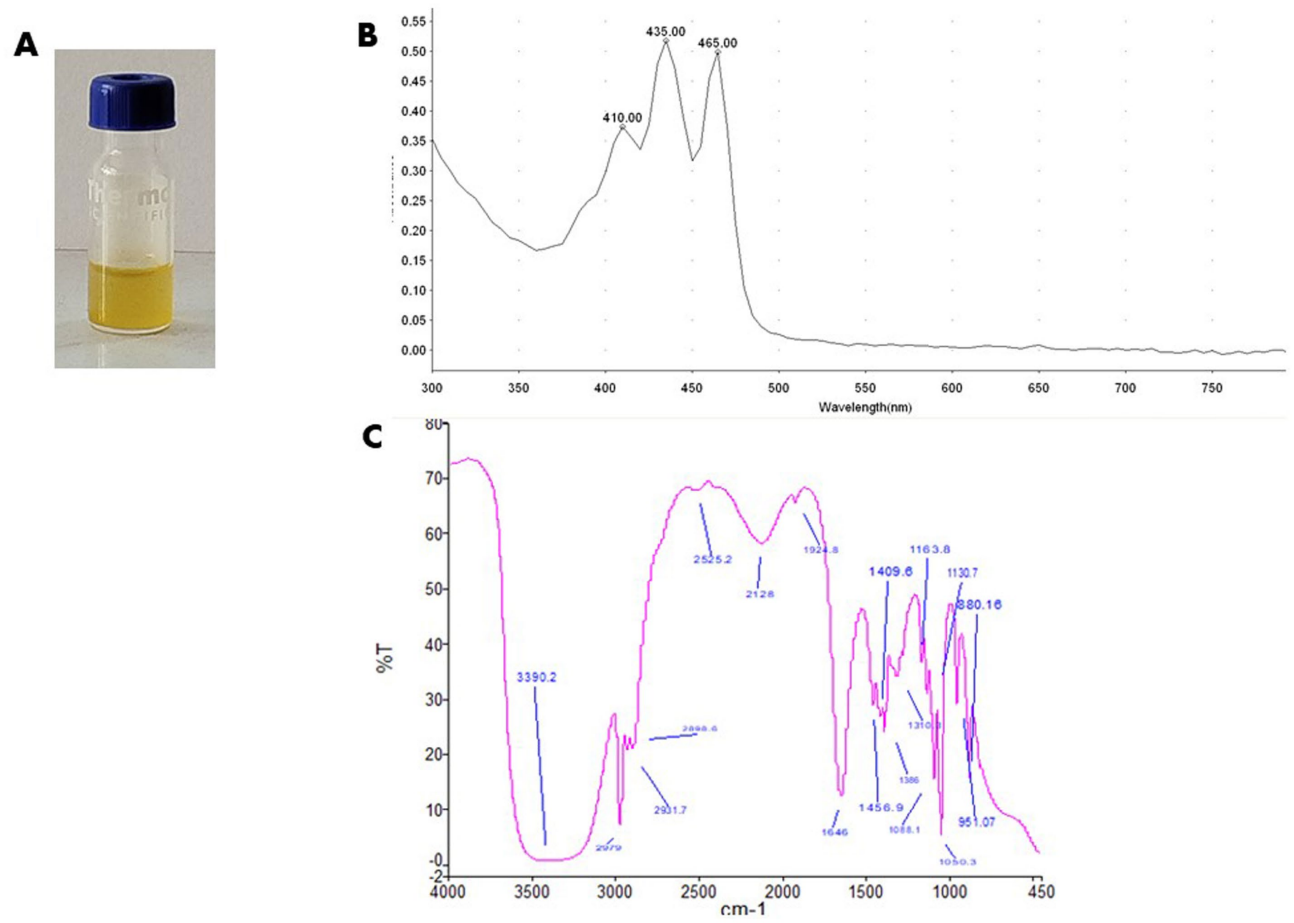
Spectral analysis of carotenoid extract from *Agrococcus* sp. NP24. **(A)** Methanolic extract of NP24 carotenoids. **(B)** UV/Vis scanning at 200–800 nm showing a characteristic three fingered chromatogram with maximum absorbance at 435 nm, FTIR spectrum showing the main functional group in the pigment.

NP24 pigment methanolic extract appeared as clear yellow liquid (Figure 2A). Pigment extract demonstrated a characteristic three fingered absorption spectrum distinctive to carotenoids class, with maximum absorbance at 435 nm (Figure 2B).

FTIR spectral analysis (Figure 2C) revealed a characteristic absorption band at 1,650 cm^−1^, attributed to C=C stretching vibrations of alkenes, indicating the presence of aliphatic groups in the carotenoid extract. Additionally, the band at 1,456 cm^−1^ corresponds to CH₂ stretching, whereas the peaks observed at 1,386 cm^−1^ and 1,159 cm^−1^ are assigned to C–H and C–OH stretching vibrations, respectively.

**Figure 3:**
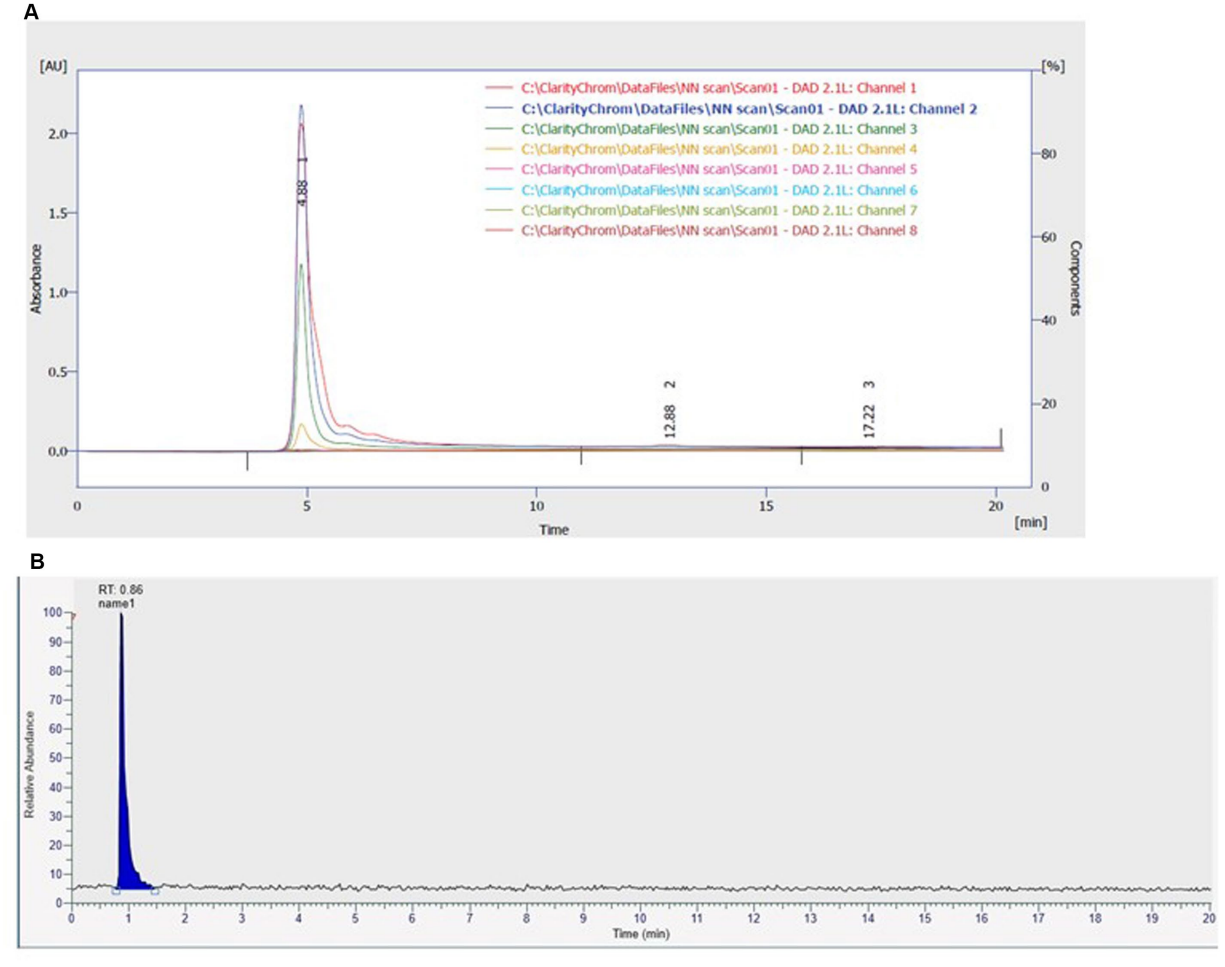
HPLC-DAD and MS chromatographic profiles of NP24 carotenoid. **(A)** HPLC-DAD chromatogram recorded across DAD channels showing a single dominant peak at RT = 4.89 min with no additional peaks detected across the 0–20 min separation. **(B)** UHPLC–MS chromatogram of NP24 showing a single peak at RT = 0.86 min.

NP24 carotenoid eluted as a single major component in HPLC-DAD, giving one sharp peak at RT = 4.89 min across the DAD channels (0–20 min run) with no additional detectable peaks. Consistently, MS analysis produced a single chromatographic peak (RT shown in the MS trace = 0.86 min) and a mass spectrum at the peak apex consistent with a carotenoid, supporting both identity and pigment purity (Figures 3A,B).

**Figure 4:**
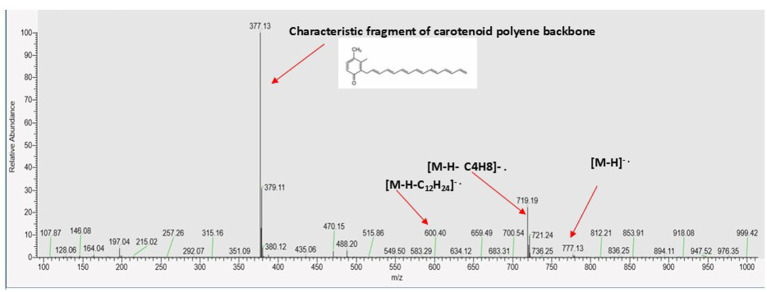
LC/ESI-MS–MS of NP 24 carotenoid extract in negative ionization mode.

The spectrum of NP24 carotenoid showed single peak on both HPLC-DAD and MS. The fragmentation pattern in negative ion mode identified the carotenoid as zeaxanthin monoester (M-C14:0) (Figure 4 and Tables 1, 2).

**Table 1:** Analysis of the HPLC chromatogram of the extracted carotenoid showing retention time (*T*_R_) and maximum absorption wavelength (*λ*_max_) of the evolved peak.

Peak number	*T*_R_ (min)	*λ*_max_	[M-H]^.-^ (m/z)	Carotenoid identification	Formula
1	4.23	435	777	Zeaxanthin monoester	M-C14:0

LC/ESI-MS/MS mass spectra of the target peak.

**Table 2:** Typical ions detected in negative ion mode (ESI-MS/MS) of *Agrococcus* sp. NP24 carotenoid component.

Fragment ions (m/z)	Formula	Reference
719 [M-H-58]^.-^	[M-H-C_4_H_8_]	Mercadante et al. (2017) and Frassanito et al. (2008)
600.4 [M-H-174]^.-^_	[M-H-C_12_H_24_]	Mercadante et al. (2017) and Frassanito et al. (2008)
377 [M-H-399]^.-^	Characteristic fragment of polyene structure	Rivera et al. (2014)

### 3.3 Pigment stability assessment

Pigment stability was evaluated under different pH conditions, temperatures, and exposure to sunlight (Table 3). The pigment remained fully stable up to 50 °C but showed a noticeable decline at higher temperatures, with approximately 50% loss after 1 h. of incubation at 70 °C. The effect of pH revealed greater stability in acidic conditions (pH 3–5) compared to neutral and alkaline ones (pH 6–9). Under alkaline conditions, the pigment was totally instable with visible precipitation under short incubation time. Exposure to sunlight adversely affected the pigment extract, leading to severe degradation after 1 h. of daylight exposure, leaving a residual concentration of 26%.

**Table 3:** The stability of NP24 carotenoids against some physical factors.

pH	*Stability %	Temperature	*Stability %	Light effect	*Stability %
3	310.5 ± 2.47	20 °C	99.0 ± 0.98	Day light	26% ± 2.5
4	112 ± 0.24	30 °C	115 ± 2.4		
5	108 ± 1.48	40 °C	104 ± 1.97		
6	63.7 ± 1.69	50 °C	102.5 ± 1.48		
7	41.5 ± 1.48	60 °C	62.5 ± 2.33		
8	ND	70 °C	52.0 ± 2.89		
9	ND				

*Methanolic extract of NP24 carotenoid stored at 4 °C in dark conditions was set as the control test.

ND: Not detected due to carotenoid precipitation under alkaline conditions.

All tests were carried out in triplicates and data are displayed as ± SE.

### 3.4 Optimization of carotenoid production on whey-based medium

**Figure 5:**
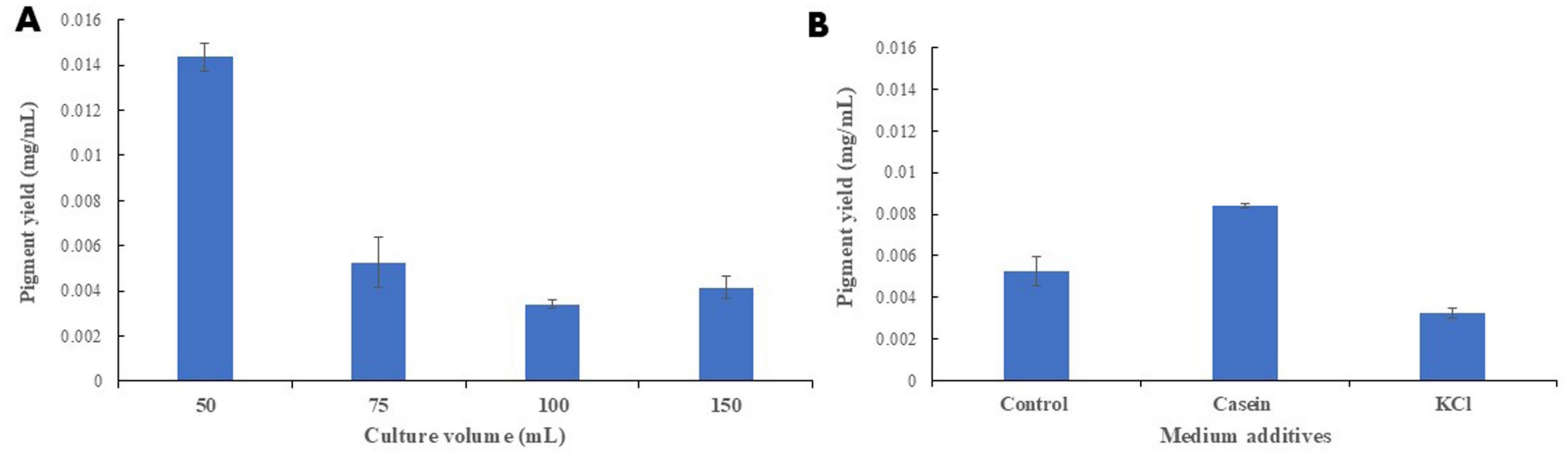
Optimizing pigment production via OVAT approach. **(A)** Testing different culture volumes on pigment yield. All trials were carried out in 250 mL Erlenmeyer flask. Seventy-five mL culture volume was set as the control trial. **(B)** Studying the effect of casein and KCl addition on pigment yield. The control trial consists of 80% (v/v) cheese whey, 1% (w/v) peptone, 0.5 g % (w/v), 0.5% (w/v) MgSO_4_. All trials were carried out in 250 mL Erlenmeyer flasks incubated at 20 °C for 3 days at 85 rpm. All trials were carried out in triplicates ± SE.

The effect of culture volume on pigment accumulation was tested via OVAT approach using different culture volumes. Reducing the working volume to 50 mL had significantly improved pigment yield with more than 3 times compared to higher volumes (statistical significance was set at *p* ≤ 0.05) (Figure 5A).

The effect of modifying the medium composition on carotenoids yield revealed a positive effect of casein while the addition of KCl had significantly reduced carotenoids production (Figure 5B). In addition, the effect of omitting MgSO_4_ and yeast extract additives had significantly lowered the total yield (data not shown). However, higher concentrations of MgSO_4_ and yeast extract negatively affected pigment accumulation (data not shown).

In this respect, a fixed concentration of MgSO4 and yeast extract was applied, while the concentrations of cheese whey (X1), peptone (X2) and casein (X3) were estimated via BBD in a 15-trial matrix at three encoded levels −1, 0, 1. Table 4 displays the designed matrix, the coded and actual level of each factor, and the response of each trial in terms of pigment concentration (mg/mL) quantified at 435 nm. Table 5 shows the coefficients, *p*-value and *t stat* of the tested variables.

**Table 4:** BBD matrix, with coded and real values along with the predicted and experimental responses, of pigment production in terms of (mg/mL).

Variables				Pigment concentration (mg/mL)		
Trial number	X1	X2	X3	Experimental	Predicted	Residual
1	0	0	0	0.0220	0.0220	-3.5E-18
2	0	0	0	0.0220	0.0220	-3.5E-18
3	0	-1	1	0.0176	0.0229	−0.00539
4	0	1	−1	0.0102	0.0048	0.00538
5	−1	−1	0	0.0175	0.0159	0.00152
6	0	0	0	0.0220	0.0220	3.5E−18
7	1	0	-1	0.0192	0.0230	−0.00386
8	0	1	1	0.0280	0.0228	0.00513
9	0	−1	−1	0.0260	0.0311	−0.00514
10	−1	0	1	0.0181	0.0142	0.00386
11	−1	0	−1	0.0116	0.0079	0.00361
12	1	0	1	0.0231	0.0267	−0.00361
13	1	1	0	0.0150	0.0165	−0.00153
14	1	−1	0	0.0650	0.0560	0.009
15	−1	1	0	0.020	0.029	−0.009

Variables	Code	Coded level and actual level		
		−1	0	1
Cheese whey (v/v)	X1	40	60	80
Peptone % (w/v)	X2	0.5	1.0	1.5
Casein % (w/v)	X3	0.6	1.0	1.4

**Table 5:** Statistical analysis of BBD showing *coefficients, t-* and *p-values* for significant variables affecting pigment production by *Agrococcus* sp. NP24.

Term	Coefficients	Standard error	*t* stat	*P*-value	Upper 95.0%
Intercept	0.022	0.004715	4.666246	0.005501	0.03412
X1	0.006888	0.002887	2.385563	0.062731	0.014309
X2	−0.00661	0.002887	−2.29031	0.070622	0.000809
X3	0.002475	0.002887	0.857244	0.430472	0.009897
X1X2	−0.01313	0.004083	−3.2145	0.02361	−0.00263
X1X3	−0.00065	0.004083	−0.15919	0.879746	0.009846
X2X3	0.00655	0.004083	1.604189	0.169578	0.017046
X1X1	0.002463	0.00425	0.579441	0.587417	0.013387
X2X2	0.004913	0.00425	1.155941	0.299935	0.015837
X3X3	−0.00646	0.00425	−1.52067	0.188823	0.004462

Model significance was inferred from *p*-value (0.105) and *F* value (3.21) displayed by ANOVA analysis (Table 6). The small regression value (*p* = 0.056) proves the accuracy of the model in elucidating the relationship between the response and the involved independent variables. The value of *R^2^* was 0.85, indicating a high degree of association between the predicted and experimental values.

**Table 6:** ANOVA analysis of BBD experimental data.

Source	df	SS	MS	*F*	Significance *F*
Regression	9	0.001928	0.000214	3.2129	0.1058
Residual	5	0.000333	6.67E-05		
Total	14	0.002262			

**Figure 6:**
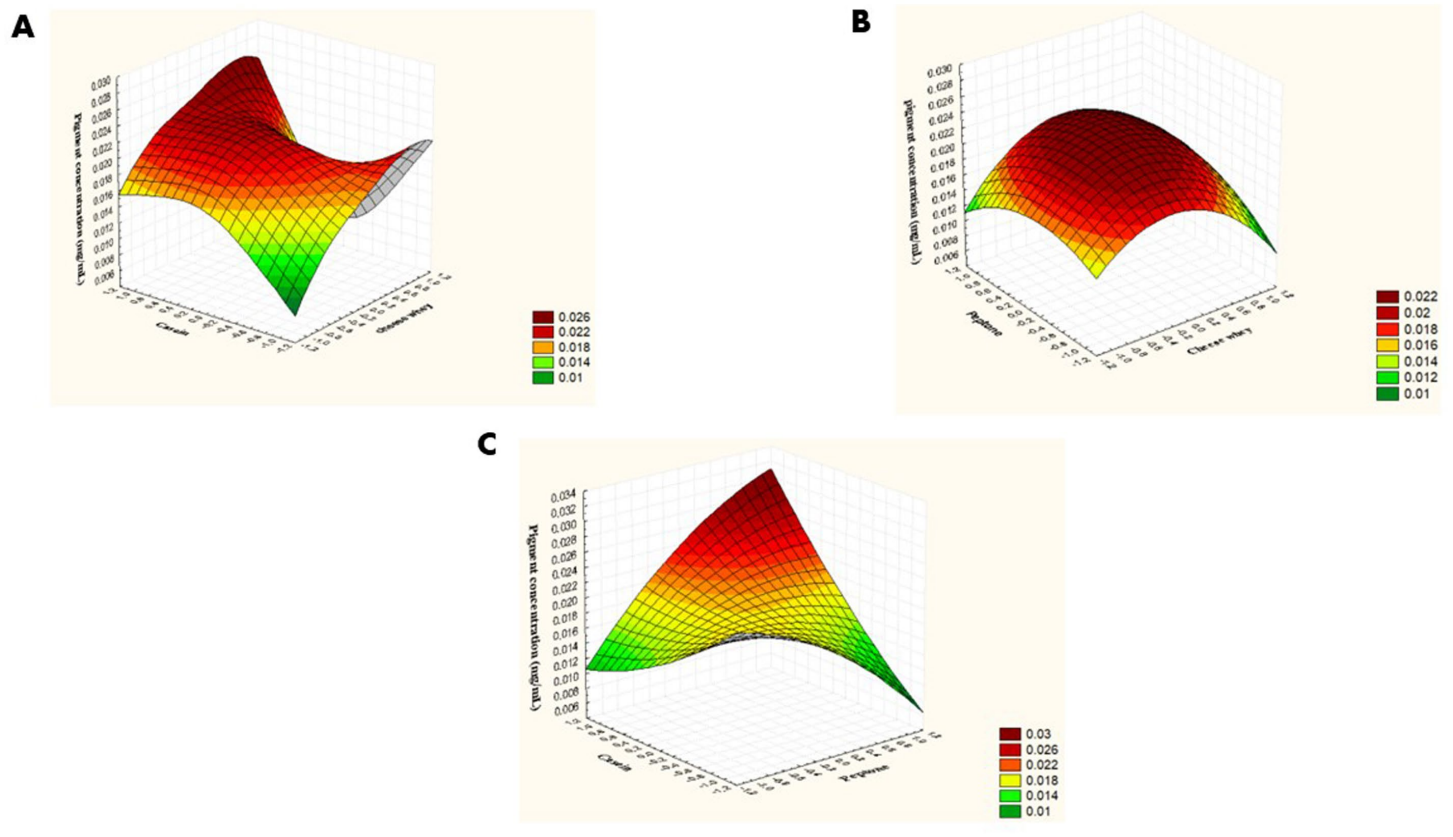
Three-dimensional surface plots showing the interactive effect between the three independent variables on pigment concentration expressed in (mg/mL). **(A)** Whey and peptone; **(B)** whey and casein; **(C)** peptone and casein.

The interactions among the variables are illustrated via Surface plots (Figure 6). The interactive effect between cheese whey and peptone is represented in Figure 6A. A significant antagonistic interaction was observed between whey and peptone (X1 × X2), as evidenced by a negative interaction coefficient (−0.013125) and a significant *p*-value (*p* = 0.02). In contrast, the interactions between whey and casein (X1 × X3) (Figure 6B) and between peptone and casein (Figure 6C) (X2 × X3) were not statistically significant (*p* = 0.879 and *p* = 0.169, respectively), indicating that pigment production in these cases was governed mainly by the individual effects of whey and peptone rather than by their interactions with casein.

The exact concentrations of each variable and the maximum response were inferred from integrating the obtained results in a second order polynomial (Equation 4), expressing all forms of interactions.


$$\begin{array}{l} Y_{\text{pigment yield}\;(\mathrm{mg}/\mathrm{mL})}=0.022+0.006888\times 1–0.00661\times 2\\ +0.00247\times 3–0131\mathrm{3X}1X2–0.0006X1\mathrm{X3}+0.0065\mathrm{5X}2\mathrm{X3}\\ +0.0024\mathrm{6X}1\mathrm{X1}+0.0049X2X2–0.0064\mathrm{6X}3\mathrm{X3} \end{array}$$ (4)

Diffracting the polynomial equation revealed the optimal value for each variable to be 80% (v/v) cheese whey, 0.5 g % (w/v) peptone and 0.97 g % (w/v) casein to achieve a maximum pigment yield of 0.0565 mg/mL.

A verification experiment was carried out with the optimal values of the three tested value to assess the model accuracy. The model achieved an accuracy of 80.1% with an experimental value of 0.0453 mg/mL compared to a predicted value of 0.056 mg/mL.

### 3.5 Biological effect of *Agrococcus* sp. NP24 carotenoid extract

#### 3.5.1 Antimicrobial effect

Upon comparing with control well (DMSO solvent), the NP24 carotenoid extract showed weak inhibitory activity with an inhibition zone of −8 mm (including 6 mm of agar well) against *E. coli*, while no detectable activity was observed against *S. aureus* and *C. albicans*.

#### 3.5.2 Antioxidant effect

**Figure 7:**
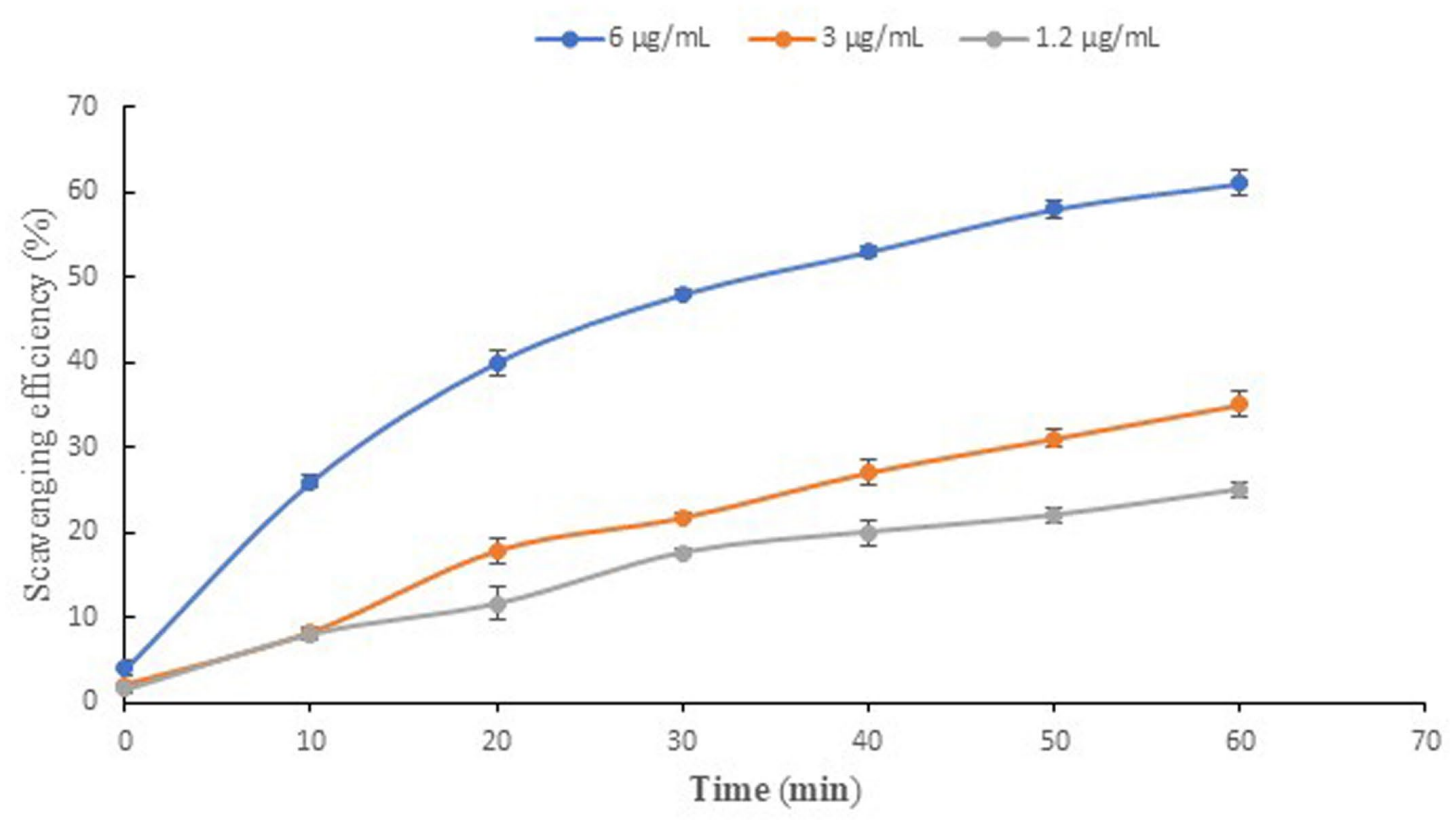
Antioxidant effect of NP24 carotenoid extract using DPPH reagents. Three concentrations of NP24 carotenoid extract were tested along 1 h. Six μg/mL achieved IC_50_ after 30 min incubation.

The three tested concentrations of NP24 carotenoids exhibited a positive correlation between carotenoid concentration and RSA%, showing increased radical scavenging activity with higher concentrations (Figure 7). The IC_50_ value was reached at a concentration of 6 μg/mL after 30 min of incubation.

#### 3.5.3 *In vitro* cytotoxic/antiproliferative effects

**Figure 8:**
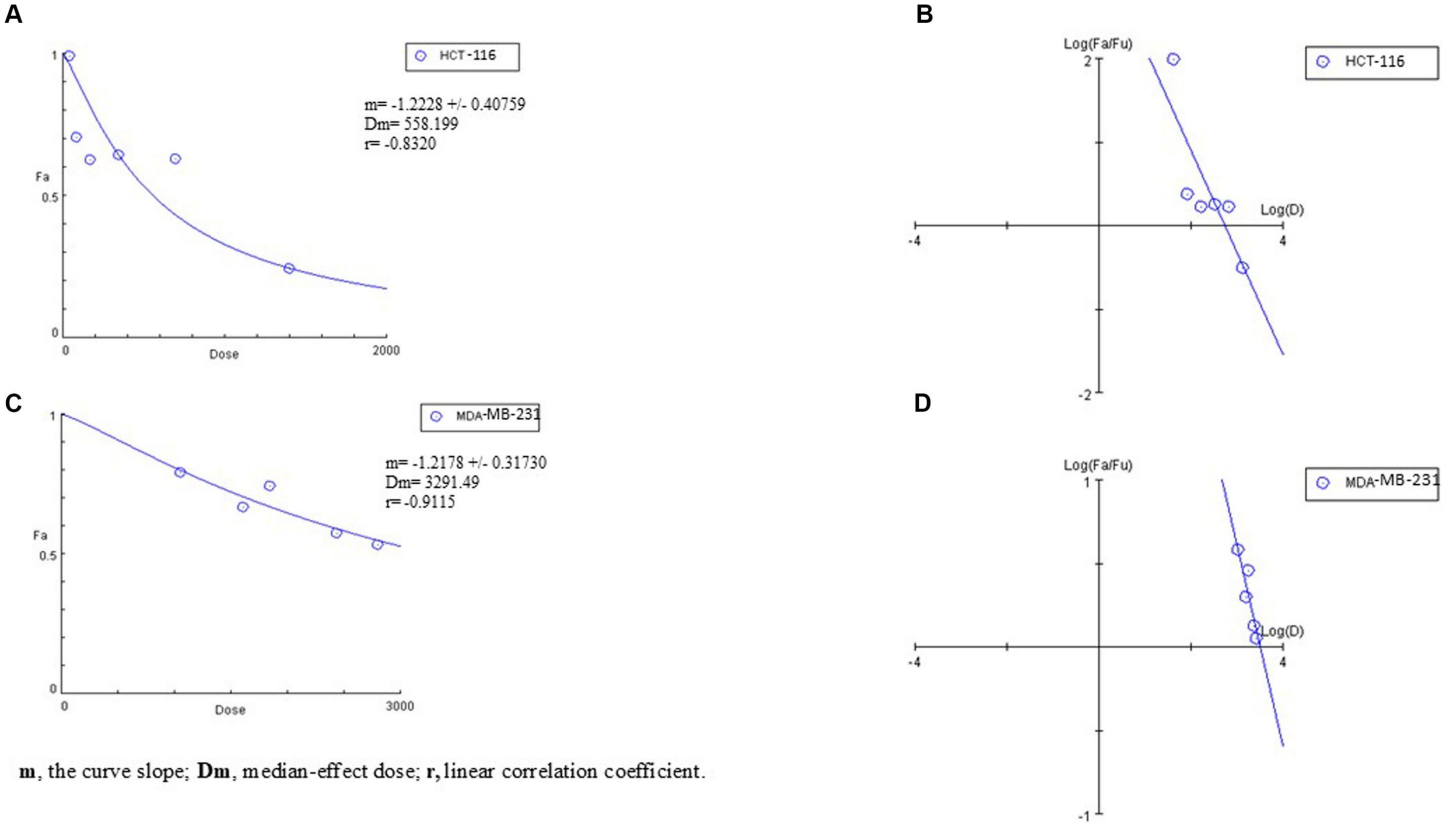
Dose–response curves **(A,C)** and median-effect plots **(B,D)** generated by CompuSyn software to estimate the IC_50_ values of the carotenoid extract against HCT-116 and MDA-MB-231 cell lines.

The anticancer activity of the carotenoid pigment produced by *Agrococcus* sp. NP24 strain was evaluated using the MTT assay after 48 h of incubation. The dose–response curves and median effect plots (Figures 8A–D) illustrate the effects of serial dilutions of the methanolic pigment extract on the viability of the tested cell lines. The IC50 values of the carotenoid extract against the TNBC cell line MDA-MB-231 and the colorectal carcinoma cell line HCT-116 were 3.3 mg/mL and 0.56 mg/mL, respectively, whereas no cytotoxic effect was observed against the hepatoblastoma cell line HepG-2. Notably, the carotenoid extract exhibited an IC50 value of approximately 33.5 mg/mL against normal PBMCs, suggesting its potential selectivity and safety for anticancer applications.

**Figure 9:**
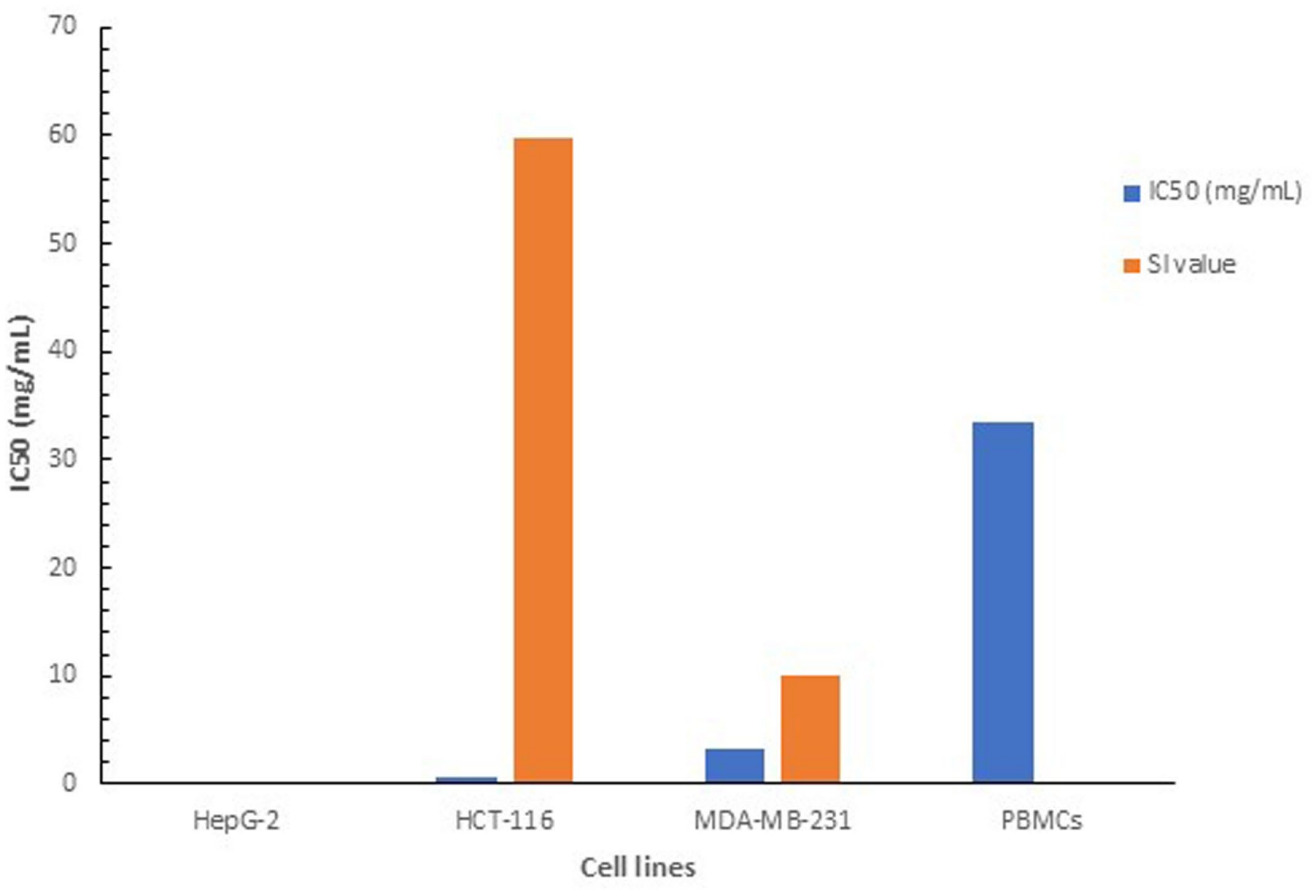
IC_50_ and SI values of the carotenoid extract of *Agrococcus* sp. NP24 determined by the MTT assay on the three tested cell lines.

The safety and selectivity of the NP24 carotenoid extract were further assessed by calculating the selectivity index (SI). The SI value measures the selective cytotoxicity of the investigated compound toward cancer cells relative to normal cells, and is calculated as the ratio of the IC50 value of the investigated compound, recorded for the PBMCs to the IC50 value recorded for the respective cell line. Accordingly, a higher SI value promises a more selective and hence safer therapeutic potential. The SI values for the MDA-MB-231 and HCT-116 cell lines were 10.2 and 59.8, respectively, indicating the high safety and selectivity of the carotenoid extract (Figure 9). According to Nogueira and do Rosário (2010), an SI value greater than 2 is generally required to ensure the safe application of a natural bioactive compound. Whereas Famuyide et al. (2019) suggested that an SI value above 1 can be considered satisfactory. The observed selectivity of the pigment extract remains valid even under more stringent selectivity criteria, including those proposed by Weerapreeyakul et al. (2012), who suggested an SI value ≥ 3, as well as more rigorous threshold of SI value ≥ 10 reported by Peña-Morán et al. (2016) for considering product safety.

## 4 Discussion

The transition toward a circular bioeconomy has become a global priority to minimize waste generation and emphasizing the sustainable use of biological resources. Production of natural pigments from microorganisms using waste substrates, align well with the principles of circular bioeconomy since many agri-food wastes can be used as nutritional substrates for cultivating pigment producing strains. This work frame will not only guarantee a sustainable production of natural pigment in a cost wise approach, but will also reduce the environmental burden caused by the miss handling of these wastes.

The present study aligns well with the circular bioeconomy approach, as it addresses the production of microbial carotenoids using an isolated pigmented bacterial strain identified as *Agrococcus* sp. NP24, cultivated on a whey-based medium. Cheese whey is a major byproduct of dairy processing industries, representing a substantial volume in the dairy sector, with global estimates ranging between 160 and 190 million tons per year (Arshad et al., 2022). However, despite this large volume, more than 40% of whey remains under-utilized or disposed, leading to both environmental and economic burdens (Ahmed et al., 2023). Because cheese whey is rich in nutrients, such as soluble proteins, lactose, minerals, and vitamins, its direct disposal poses a significant environmental challenge. This is primarily due to its high organic load, which can deplete dissolved oxygen, disrupt aquatic ecosystems, and cause eutrophication. In this context, utilizing whey as a fermentation substrate for microbial pigment production offers a promising strategy for valorizing this byproduct while simultaneously mitigating its ecological impact.

*Agrococcus* genus is classified as Actinobacteria, order Micrococcales, family Microbacteriaceae (Whitman et al., 2012). A recent study carried out by Ding et al. (2022) showed novel bioactive metabolites from an isolated marine *Agrococcus* strain such as furan fatty acid and linear tetradepsipeptide, which highlights the importance of *Agrococcus* bacteria as a potential source of new bioactive compounds. Although previous studies reported the presence of carotenoid biosynthetic genes in *Agrococcus pavilionensis* RW1 and *Agrococcus lahaulensis* K22-21 genomes, including phytoene synthase, *β*-carotene ketolase (White et al., 2018), the research on *Agrococcus* carotenoids in terms of production optimization bioactivity and evaluation is quite limited.

Different species of *Agrococcus* genus produce a number of enzymes, enabling them to metabolize a wide range of carbon sources. Although β-galactosidase activity is not common among several spp. of *Agrococcus* genus (Behrendt et al., 2008), the isolated NP24strain was able to effectively use lactose sugar as the main carbon source (data not shown), which favored the use of whey, a lactose-rich byproduct, as a suitable and sustainable substrate for microbial pigment production.

Pigment production, like most fermentation processes, is strongly influenced by the composition of the growth medium as well as the cultivation parameters such as temperature and aeration. Therefore, optimization of these conditions is important for maximizing production efficiency. Aeration represents a crucial factor in pigment synthesis. High aeration ratio can enhance the activity of oxygen-dependent enzymes involved in pigment biosynthesis (e.g., desaturases, oxidases) (Musaalbakri et al., 2006). However, in certain microorganisms limited aeration can act as a stress factor, favoring alternative pathways involved in pigment accumulation (Sarian et al., 2016). There are two factors affecting oxygen transfer; the gas–liquid interfacial area (working volume) and the agitation speed. Aeration factor had significantly affected carotenoid synthesis in NP24 strain. Where a working volume of 50 mL incubated at low rpm (85 rpm) achieved the maximum yield.

High performance liquid chromatography is the main analytical technique applied for carotenoid identification. Reversed-phase high performance liquid chromatography (RP-HPLC) is the preferred method for separating carotenoids due to its excellent selectivity and simplicity of usage (Bijttebier et al., 2013). The spectral characteristics of carotenoid can be anticipated via photodiode array detector (PDA), while molecular weight and fragmentation pattern can be identified from mass spectrometric (MS) detectors. In the HPLC-DAD chromatogram, NP24 eluted as a single dominant, symmetrical peak at RT = 4.89 min, consistently observed across the DAD channels (200, 210, 230, 254, 320, 400, 450, and 500 nm), with no additional detectable peaks over the 0–20 min run. In the UHPLC–MS chromatogram, NP24 likewise produced a single chromatographic peak (RT = 0.86 min in the MS trace shown), and the mass spectrum acquired at the peak apex corresponded to the expected carotenoid molecular ion with characteristic fragment ions (Table 2), supporting compound identity. Together, the presence of a single peak in both DAD and MS (and the absence of secondary peaks) indicates that the isolated pigment is of high purity under the applied chromatographic conditions.

Esterified carotenoids are derivatives of xanthophyll that have one or both hydroxyl groups bound to fatty acids by ester bonds. This modification boosts the hydrophobicity and stability of carotenoids; it also contributes in protecting the polyene chain from oxidation and isomerization. Carotenoid esterification usually found in natural sources such as certain microorganisms, fruits and flowers, functioning as stabilized variant and storage form of free xanthophylls like lutein, zeaxanthin and violaxanthin. In mass spectrometric analysis, esterified xanthophylls usually show the molecular ion of the intact ester in addition to characteristic fragment ions corresponding to the cleavage of the fatty acid moiety (e.g., neutral loss of an acyl group) and/or the carotenoid backbone (Mercadante et al., 2017; Frassanito et al., 2008). The molecular ion peak of the separated peak [M-H]-^.^ was 777 corresponding to zeaxanthin monoester (C14:0). A fragment at m/z 719 [M-H-58]^-.^, represents a loss of small fraction, plausibly a short alkyl group (C_4_H_8_). The ester degradation sequence was confirmed by a fragment ion at m/z 600, which represents a larger cleavage of the alkyl group (C_12_H_24_). In addition, a characteristic ion peak of carotenoids polyene chain degradation was detected at m/z 377 (Rivera et al., 2014).

Carotenoids generally exhibit varied stability under different physical conditions, mainly due to their long polyene chains, which are highly susceptible to degradation by factors such as pH, temperature, and light. In general, carotenes display a higher temperature stability profile than xanthophylls, owing to their lack of oxygen-containing functional groups and their purely hydrocarbon backbones. However, the complete stability of the NP24 pigment up to 50 °C is consistent with the previously reported behavior of esterified xanthophylls, which typically exhibit greater structural stability and enhanced resistance to thermal degradation compared to their free counterparts (Moura et al., 2023). The thermal stability of carotenoids is an advantageous property for food applications, as it helps preserve their nutritional and functional value during processing and storage (Meléndez-Martínez et al., 2023). Like most carotenoids, NP24 pigment showed better stability at acidic conditions compared to neutral one by increasing the absorbance by about 310, 112, 108% at pH 3, 4 and 5, respectively (Cheng et al., 2023). The enhancement in absorbance can be explained by increased pigment solubility and absence of hydroxide ions. However, the pigment completely precipitated at alkaline conditions which can be attributed to the hydroxide-induced oxidation and decreased solubility (Cheng et al., 2023).

The low photostability of the tested carotenoid matches with the well-known photosensitivity of carotenoids. Where, the conjugated double bond system makes them highly reactive toward free radicals generated by light, causing isomerization and photooxidation under light exposure (Aslam et al., 2021).

The biological activity of natural pigments such as antioxidant, anticancer, antimicrobial usually helps in understanding their possible applications in pharmaceutical, nutraceutical, or food industries. NP24 zeaxanthin monoester extract showed anticancer activity against two cancer cell lines with variable potency. The production of carotenoids with anticancer properties by various bacterial taxa has been documented in several studies. A previous study by Choi et al. (2014) reported the synthesis of deinoxanthin by the radioresistant actinomycete *Deinococcus radiodurans*, with IC₅₀ values of 59, 61, and 77 μM against the HepG-2, HT-29, and PC − 3 cell lines, respectively. Similarly, (Afra et al., 2017) reported the anticancer activity of red carotenoid-related pigments from a marine *Arthrobacter* sp. against the oesophageal cancer cell line KYSE30, with an IC₅₀ value of 23.5 μg/mL. The same pigment also exhibited antioxidant potential in the DPPH assay, with an IC₅₀ value of 4.5 mg/mL. Later, Tapia et al. (2021) demonstrated that the extremophilic actinomycete *Deinococcus* sp. UDEC-P1, isolated from Patagonia, produced deinoxanthin with selective anticancer activity against the human osteosarcoma cell line Saos-2. In contrast, *Arthrobacter* sp. UDEC-P1, isolated from Antarctica, synthesized other carotenoids that exhibited selective antiproliferative effects against the cancer cell lines MCF-7 (human breast adenocarcinoma), Saos-2 (human osteosarcoma), and Neuro-2a (mouse neuroblastoma).

In this work, human cancer cell lines were selected to represent clinically distinct carcinoma models to evaluate the broad-spectrum anticancer potential of the bacterial carotenoids across diverse types of carcinomas. Considering that the anti-colorectal activity previously reported in the literature for bacterial carotenoids was directed mostly to HT-29 human colorectal carcinoma cells (Choi et al., 2014); however, more cell lines modeling distinct genetic backgrounds of colorectal carcinoma remain unexplored; hence, in the current study it was aimed to investigate the anticancer activity of *Agrococcus* sp. carotenoids against HCT-116 cells known to be a highly proliferative, tumorigenic, and poorly differentiated epithelial subtype of colorectal carcinoma, harboring a key mutation in codon 13 of KRAS proto-oncogene (Rajput et al., 2008) rendering HCT-116 cell line commonly used model in drug screening studies. Similarly, MDA-MB-231 cells were selected to model a highly dedifferentiated, mesenchymal, basal subtype of human TNBC which lacks the expression of estrogen, progesterone, as well as HER-2 receptors; thus, is unresponsive to hormonal and HER-2 targeted therapies; consequently, MDA-MB-231 cells represents a highly aggressive, metastatic human breast adenocarcinoma associated with the high recurrence rates and poor prognosis (Chavez et al., 2011). In the same context, HepG-2 cells, earlier described as hepatocellular carcinoma, were selected to represent a well-differentiated human hepatoblastoma model (López-Terrada et al., 2009). Interestingly, the anticancer activity reported in the literature for carotenoids against the colorectal carcinoma HCT-116 cell line is mostly related to plant and algal carotenoids not bacterial (Baeza-Morales et al., 2024; Hormozi and Baharvand, 2024). Contextually, the current work is among the few reports accentuating the anti-HCT-116 activity of bacterial carotenoids which have been more commonly reported for their anti-colorectal activity against HT-29 cell lines as for the study by Choi et al. (2014) on deinoxanthin from *Deinococcus radiodurans* against HT-29 cell line.

The NP24 pigment showed selective weak antimicrobial activity only against *Escherichia coli*. Such species-specific variation has also been reported in other carotenoid studies (Naisi et al., 2023), where antimicrobial activity depended more on cell membrane interactions, pigment composition and solubility than on bacterial Gram type (Saubenova et al., 2024). Carotenoids are generally described as antioxidants and photoprotective agents, not strong antimicrobial agents. A previous study on fucoxanthin carotenoids reported only weak antibacterial effects with a median inhibition zone of 10.5 and 8.5 for Gram negative and Gram positive, respectively at a concentration of 25 μg (Karpiński and Adamczak, 2019). The weak and selective inhibition suggests that the pigment’s primary biological role may not be antimicrobial but rather associated with antioxidative mechanisms.

## Data Availability

The datasets presented in this study can be found in online repositories. The names of the repository/repositories and accession number(s) can be found in the article/supplementary material.

## References

[ref1] Afra S. Makhdoumi A. Matin M. Feizy J. (2017). A novel red pigment from marine Arthrobacter sp. G20 with specific anticancer activity. J. Appl. Microbiol. 123, 1228–1236. doi: 10.1111/jam.13576, 28862783

[ref2] Afroz Toma M. Rahman M. H. Rahman M. S. Arif M. Nazir K. N. H. Dufossé L. (2023). Fungal pigments: carotenoids, riboflavin, and polyketides with diverse applications. J. Fungi 9:454. 10.3390/jof9040454 PMC10141606 37108908

[ref3] Ahmed T. Sabuz A. A. Mohaldar A. Fardows H. M. S. Inbaraj B. S. Sharma M. . (2023). Development of novel whey-mango based mixed beverage: effect of storage on physicochemical, microbiological, and sensory analysis. Foods 12:237. doi: 10.3390/foods12020237, 36673328 PMC9858226

[ref4] Ali H. (2010). Biodegradation of synthetic dyes—a review. Water Air Soil Pollut. 213, 251–273. doi: 10.1007/s11270-010-0382-4

[ref5] Alnuqaydan A. M. (2024). The dark side of beauty: an in-depth analysis of the health hazards and toxicological impact of synthetic cosmetics and personal care products. Front. Public Health 12:1439027. doi: 10.3389/fpubh.2024.1439027, 39253281 PMC11381309

[ref6] Arshad U.-E.-T. Hassan A. Ahmad T. Naeem M. Chaudhary M. T. Abbas S. Q. . (2022). A recent glance on the valorisation of cheese whey for industrial prerogative: high-value-added products development and integrated reutilising strategies. Int. J. Food Sci. Technol. 58, 2001–2013.

[ref7] Aslam S. Ahmad M. Riaz M. (2021). “Stability of carotenoids” in Carotenoids: structure and function in the human body. eds. Zia-Ul-Haq M. Dewanjee S. Riaz M. (Cham: Springer International Publishing).

[ref8] Baeza-Morales A. Medina-Garcia M. Martinez-Peinado P. Pascual-Garcia S. Pujalte-Satorre C. Lopez-Jaen A. B. . (2024). The antitumour mechanisms of carotenoids: a comprehensive review. Antioxidants 13:1060. doi: 10.3390/antiox13091060, 39334719 PMC11428676

[ref9] Banu J. R. Kavitha S. Tyagi V. K. Gunasekaran M. Karthikeyan O. P. Kumar G. (2021). Lignocellulosic biomass based biorefinery: a successful platform towards circular bioeconomy. Fuel 302:121086. doi: 10.1016/j.fuel.2021.121086

[ref10] Barciela P. Perez-Vazquez A. Prieto M. (2023). Azo dyes in the food industry: features, classification, toxicity, alternatives, and regulation. Food Chem. Toxicol. 178:113935. doi: 10.1016/j.fct.2023.113935, 37429408

[ref11] Barreto J. V. D. O. Casanova L. M. Junior A. N. Reis-Mansur M. C. P. P. Vermelho A. B. (2023). Microbial pigments: major groups and industrial applications. Microorganisms 11:2920. doi: 10.3390/microorganisms11122920, 38138065 PMC10745774

[ref12] Behrendt U. Schumann P. Ulrich A. (2008). *Agrococcus versicolor* sp. nov., an actinobacterium associated with the phyllosphere of potato plants. Int. J. Syst. Evol. Microbiol. 58, 2833–2838. doi: 10.1099/ijs.0.2008/001610-0, 19060068

[ref13] Bijttebier S. K. D'hondt E. Hermans N. Apers S. Voorspoels S. (2013). Unravelling ionization and fragmentation pathways of carotenoids using orbitrap technology: a first step towards identification of unknowns. J. Mass Spectrom. 48, 740–754. 23722965 10.1002/jms.3203

[ref14] Boyum A. (1968). Isolation of mononuclear cells and granulocytes from human blood. Scand. J. Clin. Lab. Invest. 21, 77–89. 4179068

[ref15] Chavez K. J. Garimella S. V. Lipkowitz S. (2011). Triple negative breast cancer cell lines: one tool in the search for better treatment of triple negative breast cancer. Breast Dis. 32, 35–48. doi: 10.3233/bd-2010-0307, 21778573 PMC3532890

[ref16] Cheng W. Xian F. Zhou Z. Hu K. Gao J. (2023). Solubility and stability of carotenoids in ammonium- and phosphonium-based ionic liquids: effect of solvent nature, temperature and water. Molecules 28:3618. doi: 10.3390/molecules28083618, 37110853 PMC10143741

[ref17] Choi Y.-J. Hur J.-M. Lim S. Jo M. Kim D. H. Choi J.-I. (2014). Induction of apoptosis by deinoxanthin in human cancer cells. Anticancer Res. 34, 1829–1835, 24692716

[ref18] Chou T. Martin N. (2005). Compusyn for drug combinations and for general dose-effect Analysis. Pc Software for quantization of synergism and antagonism and determination of Ic50, Ed50 and Ld50. Paramus (NJ): ComboSyn. CompuSyn Software ComboSyn Inc.

[ref19] Ding W. Li Y. Tian X. Chen M. Xiao Z. Chen R. . (2022). Investigation on metabolites in structural diversity from the deep-sea sediment-derived bacterium *Agrococcus* sp. Scsio 52902 and their biosynthesis. Mar. Drugs 20:431. doi: 10.3390/md20070431, 35877724 PMC9323897

[ref20] Famuyide I. M. Aro A. O. Fasina F. O. Eloff J. N. Mcgaw L. J. (2019). Antibacterial and antibiofilm activity of acetone leaf extracts of nine under-investigated south African *Eugenia* and *Syzygium* (Myrtaceae) species and their selectivity indices. BMC Complement. Altern. Med. 19:141. doi: 10.1186/s12906-019-2547-z, 31221162 PMC6587284

[ref21] Flegler A. Lipski A. (2021). The C50 carotenoid bacterioruberin regulates membrane fluidity in pink-pigmented *Arthrobacter* species. Arch. Microbiol. 204:70. doi: 10.1007/s00203-021-02719-3, 34951666 PMC8709818

[ref22] Frassanito R. Cantonati M. Flaim G. Mancini I. Guella G. (2008). A new method for the identification and the structural characterisation of carotenoid esters in freshwater microorganisms by liquid chromatography/electrospray ionisation tandem mass spectrometry. Rapid Commun. Mass Spectrom. 22, 3531–3539. doi: 10.1002/rcm.3761, 18853402

[ref23] Fried R. Oprea I. Fleck K. Rudroff F. (2022). Biogenic colourants in the textile industry–a promising and sustainable alternative to synthetic dyes. Green Chem. 24, 13–35. doi: 10.1039/d1gc02968a

[ref24] Grewal J. Wołacewicz M. Pyter W. Joshi N. Drewniak L. Pranaw K. (2022). Colorful treasure from agro-industrial wastes: a sustainable chassis for microbial pigment production. Front. Microbiol. 13:832918. 35173704 10.3389/fmicb.2022.832918 PMC8841802

[ref25] Hilares R. T. De Souza R. A. Marcelino P. F. Da Silva S. S. Dragone G. Mussatto S. I. . (2018). Sugarcane bagasse hydrolysate as a potential feedstock for red pigment production by *Monascus ruber*. Food Chem. 245, 786–791. doi: 10.1016/j.foodchem.2017.11.111 29287441

[ref26] Hormozi M. Baharvand P. (2024). Astaxanthin's impact on colorectal cancer: examining apoptosis, antioxidant enzymes, and gene expression. Open Biochem. J. 18:e1874091X328849.

[ref27] Karpiński T. M. Adamczak A. (2019). Fucoxanthin-an antibacterial carotenoid. Antioxidants 8:239. doi: 10.3390/antiox8080239, 31344844 PMC6720875

[ref28] Khanna M. Zilberman D. Hochman G. Basso B. (2024). An economic perspective of the circular bioeconomy in the food and agricultural sector. Commun Earth Environ 5:507. doi: 10.1038/s43247-024-01663-6

[ref29] Kirti K. Amita S. Priti S. Mukesh Kumar A. Jyoti S. (2014). Colorful world of microbes: carotenoids and their applications. Adv. Biol. 2014, 1–13. doi: 10.1155/2014/837891

[ref30] Li M. Zhu T. Yang R. Wang Z. Liu M. Yang J. (2023). Carotenoids synthesis affects the salt tolerance mechanism of *Rhodopseudomonas palustris*. Front. Microbiol. 14:1292937. doi: 10.3389/fmicb.2023.1292937, 38075924 PMC10702980

[ref31] Liaaen-Jensen S. Jensen A. (1971). “[56] quantitative determination of carotenoids in photosynthetic tissues” in Methods in enzymology (New York, London: Elsevier, Academic Press).

[ref32] López-Terrada D. Cheung S. W. Finegold M. J. Knowles B. B. (2009). Hep G2 is a hepatoblastoma-derived cell line. Hum. Pathol. 40:1512. doi: 10.1016/j.humpath.2009.07.003, 19751877

[ref33] Lu X. Li W. Wang Q. Wang J. Qin S. (2023). Progress on the extraction, separation, biological activity, and delivery of natural plant pigments. Molecules 28:5364. doi: 10.3390/molecules28145364, 37513236 PMC10385551

[ref34] Maoka T. (2020). Carotenoids as natural functional pigments. J. Nat. Med. 74, 1–16. doi: 10.1007/s11418-019-01364-x, 31588965 PMC6949322

[ref35] Marinova G. Batchvarov V. (2011). Evaluation of the methods for determination of the free radical scavenging activity by Dpph. Bulg. J. Agric. Sci. 17, 11–24.

[ref36] Meléndez-Martínez A. J. Esquivel P. Rodriguez-Amaya D. B. (2023). Comprehensive review on carotenoid composition: transformations during processing and storage of foods. Food Res. Int. 169:112773. doi: 10.1016/j.foodres.2023.112773, 37254377

[ref37] Mercadante A. Z. Rodrigues D. B. Petry F. C. Mariutti L. R. B. (2017). Carotenoid esters in foods – a review and practical directions on analysis and occurrence. Food Res. Int. 99, 830–850. doi: 10.1016/j.foodres.2016.12.018, 28847421

[ref38] Mohan S. V. Nikhil G. N. Chiranjeevi P. Reddy C. N. Rohit M. Kumar A. N. . (2016). Waste biorefinery models towards sustainable circular bioeconomy: critical review and future perspectives. Bioresour. Technol. 215, 2–12. 27068056 10.1016/j.biortech.2016.03.130

[ref39] Molaae N. Mosayebi G. Pishdadian A. Ejtehadifar M. Ganji A. (2017). Evaluating the proliferation of human PeripheralBlood mononuclear cells using Mtt assay. Int. J. Basic Sci. Med. 2, 25–28. doi: 10.15171/ijbsm.2017.06

[ref40] Moura J. D. S. Sousa R. P. E. Martins L. H. D. S. Costa C. E. F. D. Chisté R. C. Lopes A. S. (2023). Thermal degradation of carotenoids from Jambu leaves (*Acmella oleracea*) during convective drying. Foods 12:1452. doi: 10.3390/foods12071452, 37048271 PMC10093540

[ref41] Musaalbakri A. Ariff A. Rosfarizan M. Ismail A. (2006). Aeration and agitation strategies for the improvement of red pigment production by *Monascus purpureus* Ftc 5391. J. Trop. Agric. Food Sci. 34:89.

[ref42] Naisi S. Bayat M. Zahraei Salehi T. Rahimian Zarif B. Yahyaraeyat R. (2023). Antimicrobial and anti-biofilm effects of carotenoid pigment extracted from *Rhodotorula glutinis* strain on food-borne bacteria. Iran J Microbiol 15, 79–88. doi: 10.18502/ijm.v15i1.11922, 37069901 PMC10105281

[ref43] Negi A. (2025). Natural dyes and pigments: sustainable applications and future scope. Sustain. Chem. 6:23. doi: 10.3390/suschem6030023

[ref44] Noby N. Khattab S. N. Soliman N. A. (2023). Sustainable production of bacterioruberin carotenoid and its derivatives from *Arthrobacter agilis* Np20 on whey-based medium: optimization and product characterization. Bioresour. Bioprocess. 10:46. doi: 10.1186/s40643-023-00662-3, 38647623 PMC10991996

[ref45] Nogueira F. Do Rosário V. E. (2010). Methods for assessment of antimalarial activity in the different phases of the *Plasmodium* life cycle. Revista Pan-Amazônica Saúde 1, 15–15.

[ref46] Pati S. Sarkar T. (2022). Biomass conversion and biorefinery. Berlin Heidelberg, Germany: Springer.

[ref47] Pelz A. Wieland K.-P. Putzbach K. Hentschel P. Albert K. Götz F. (2005). Structure and biosynthesis of staphyloxanthin from *Staphylococcus aureus**. J. Biol. Chem. 280, 32493–32498. doi: 10.1074/jbc.m505070200 16020541

[ref48] Peña-Morán O. A. Villarreal M. L. Álvarez-Berber L. Meneses-Acosta A. Rodríguez-López V. (2016). Cytotoxicity, post-treatment recovery, and selectivity analysis of naturally occurring podophyllotoxins from *Bursera fagaroides var. fagaroides* on breast cancer cell lines. Molecules 21:1013. doi: 10.3390/molecules21081013, 27527135 PMC6274026

[ref49] Perez C. (1990). Antibiotic assay by agar-well diffusion method. Acta Biol. Med. Exp. 15, 113–115.

[ref50] Pescuma M. De Valdez G. F. Mozzi F. (2015). Whey-derived valuable products obtained by microbial fermentation. Appl. Microbiol. Biotechnol. 99, 6183–6196. doi: 10.1007/s00253-015-6766-z, 26124070

[ref51] Rajput A. San Martin I. D. Rose R. Beko A. Levea C. Sharratt E. . (2008). Characterization of Hct116 human colon cancer cells in an orthotopic model. J. Surg. Res. 147, 276–281. doi: 10.1016/j.jss.2007.04.021, 17961596

[ref52] Reis-Mansur M. C. P. P. Cardoso-Rurr J. S. Silva J. V. M. A. De Souza G. R. Cardoso V. D. S. Mansoldo F. R. P. . (2019). Carotenoids from Uv-resistant Antarctic *Microbacterium* sp. lemmj01. Sci. Rep. 9:9554. 31266976 10.1038/s41598-019-45840-6 PMC6606617

[ref53] Reji M. Kumar R. (2022). Response surface methodology (Rsm): an overview to analyze multivariate data. Indian J. Microbiol. Res. 9, 241–248.

[ref54] Rivera S. M. Christou P. Canela-Garayoa R. (2014). Identification of carotenoids using mass spectrometry. Mass Spectrom. Rev. 33, 353–372. 24178708 10.1002/mas.21390

[ref55] Saenge C. Cheirsilp B. Suksaroge T. T. Bourtoom T. (2011). Potential use of oleaginous red yeast *Rhodotorula glutinis* for the bioconversion of crude glycerol from biodiesel plant to lipids and carotenoids. Process Biochem. 46, 210–218. doi: 10.1016/j.procbio.2010.08.009

[ref56] Sarian F. D. Rahman D. Y. Schepers O. Van Der Maarel M. J. E. C. (2016). Effects of oxygen limitation on the biosynthesis of photo pigments in the red microalgae *Galdieria sulphuraria* strain 074G. PLoS One 11:e0148358. 26859750 10.1371/journal.pone.0148358 PMC4747464

[ref57] Saubenova M. Rapoport A. Venkatachalam M. Dufossé L. Yermekbay Z. Oleinikova Y. (2024). Production of carotenoids by microorganisms. Fermentation 10:502. doi: 10.3390/fermentation10100502

[ref58] Schalchli H. Hormazábal E. Astudillo Á. Briceño G. Rubilar O. Diez M. C. (2021). Bioconversion of potato solid waste into antifungals and biopigments using *Streptomyces* spp. PLoS One 16:e0252113. doi: 10.1371/journal.pone.0252113, 34019577 PMC8139487

[ref59] Siziya I. N. Hwang C. Y. Seo M. J. (2022). Antioxidant potential and capacity of microorganism-sourced C(30) carotenoids-a review. Antioxidants 11:1963. doi: 10.3390/antiox11101963, 36290686 PMC9598406

[ref60] Taha A. Gouda S. (2025). Eco-friendly dye removal: impact of dyes on aquatic and human health and sustainable fungal treatment approaches. Egypt. J. Aquat. Biol. Fish. 29, 2733–2763.

[ref61] Tapia C. López B. Astuya A. Becerra J. Gugliandolo C. Parra B. . (2021). Antiproliferative activity of carotenoid pigments produced by extremophile bacteria. Nat. Prod. Res. 35, 4638–4642. doi: 10.1080/14786419.2019.1698574, 31809588

[ref62] Vieira Rubio F. T. Maciel G. M. Bortolini D. G. De Andrade Arruda Fernandes I. Pedro A. C. Ribeiro I. S. . (2025). Artificial dyes: health risks, environmental concerns, and the rise of natural alternatives. Trends Food Sci. Technol. 162:105085. doi: 10.1016/j.tifs.2025.105085

[ref63] Vijayarathna S. Sasidharan S. (2012). Cytotoxicity of methanol extracts of *Elaeis guineensis* on Mcf-7 and Vero cell lines. Asian Pac. J. Trop. Biomed. 2, 826–829. doi: 10.1016/S2221-1691(12)60237-8, 23569855 PMC3609226

[ref64] Vural Gursel I. Elbersen B. Meesters K. P. H. Van Leeuwen M. (2022). Defining circular economy principles for biobased products. Sustainability 14:12780. doi: 10.3390/su141912780

[ref65] Weerapreeyakul N. Nonpunya A. Barusrux S. Thitimetharoch T. Sripanidkulchai B. (2012). Evaluation of the anticancer potential of six herbs against a hepatoma cell line. Chin. Med. 7:15. doi: 10.1186/1749-8546-7-15, 22682026 PMC3502167

[ref66] Weisburg W. G. Barns S. M. Pelletier D. A. Lane D. J. (1991). 16S ribosomal DNA amplification for phylogenetic study. J. Bacteriol. 173, 697–703. doi: 10.1128/jb.173.2.697-703.1991, 1987160 PMC207061

[ref9001] Whitman W. B. Goodfellow M. Kämpfer P. Busse H. J. Trujillo M. E. Ludwig W. (2012). The actinobacteria. BMSB. 5,1–2028., 30374333

[ref67] White R. A. Gavelis G. Soles S. A. Gosselin E. Slater G. F. Lim D. S. . (2018). The complete genome and physiological analysis of the microbialite-dwelling *Agrococcus pavilionensis* sp. nov; reveals genetic promiscuity and predicted adaptations to environmental stress. Front. Microbiol. 9:2180. doi: 10.3389/fmicb.2018.02180, 30374333 PMC6196244

[ref68] Yaashikaa P. R. Kumar P. S. Varjani S. (2022). Valorization of agro-industrial wastes for biorefinery process and circular bioeconomy: a critical review. Bioresour. Technol. 343:126126. doi: 10.1016/j.biortech.2021.126126, 34673193

[ref69] Zaky A. S. Kumar S. Welfle A. J. (2022). “Integrated approaches and future perspectives” in Waste-to-energy: recent developments and future perspectives towards circular economy. eds. Abomohra A. E.-F. Wang Q. Huang J. (Cham: Springer International Publishing).

